# Interaction of extracellular S100A4 with RAGE prompts prometastatic activation of A375 melanoma cells

**DOI:** 10.1111/jcmm.12808

**Published:** 2016-03-01

**Authors:** Nadine Herwig, Birgit Belter, Susann Wolf, Cathleen Haase‐Kohn, Jens Pietzsch

**Affiliations:** ^1^ Department of Radiopharmaceutical and Chemical BiologyInstitute of Radiopharmaceutical Cancer ResearchHelmholtz‐Zentrum Dresden‐RossendorfDresdenGermany; ^2^ Department of Chemistry and Food ChemistryTechnische Universität DresdenDresdenGermany

**Keywords:** calcium‐binding proteins, cancer metastasis, ER‐Golgi‐dependent secretion pathway, S100 protein secretion, soluble receptor for advanced glycation endproducts

## Abstract

S100A4, a member of the S100 protein family of EF‐hand calcium‐binding proteins, is overexpressed in various tumour entities, including melanoma, and plays an important role in tumour progression. Several studies in epithelial and mesenchymal tumours revealed a correlation between extracellular S100A4 and metastasis. However, exact mechanisms how S100A4 stimulates metastasis in melanoma are still unknown. From a pilot experiment on baseline synthesis and secretion of S100A4 in human melanoma cell lines, which are in broad laboratory use, A375 wild‐type cells and, additionally, newly generated A375 cell lines stably transfected with human S100A4 (A375‐hS100A4) or human receptor for advanced glycation endproducts (A375‐hRAGE), were selected to investigate the influence of extracellular S100A4 on cell motility, adhesion, migration and invasion in more detail. We demonstrated that A375 cells actively secrete S100A4 in the extracellular space *via* an endoplasmic reticulum‐Golgi‐dependent pathway. S100A4 overexpression and secretion resulted in prometastatic activation of A375 cells. Moreover, we determined the influence of S100A4‐RAGE interaction and its blockade on A375, A375‐hS100A4, A375‐hRAGE cells, and showed that interaction of RAGE with extracellular S100A4 contributes to the observed activation of A375 cells. This investigation reveals additional molecular targets for therapeutic approaches aiming at blockade of ligand binding to RAGE or RAGE signalling to inhibit melanoma metastasis.

## Introduction

Malignant melanoma is an aggressive and treatment‐resistant malignancy of melanocytes 1, 2. In the last decades the world‐wide number of patients increased so that malignant melanoma today is responsible for about 3% of all malignant neoplasms. Among factors supposed to play a crucial role in promoting (pro)metastatic phenotypes in various cancer entities is the small acidic calcium‐binding EF‐hand protein S100A4 2, 3. The (pro)metastatic action of S100A4, in particular, can be attributed to its intracellular interaction with cytoskeletal proteins, the tumour suppressor p53 or regulation of proteins involved in remodelling of the extracellular matrix 4. Explicitly referring to melanoma, there also is clinical evidence resulting from the observation that S100A4 was present in 78% of malignant melanoma patients biopsies, however, this investigation did not differentiate between intra‐ and extracellular S100A4 5. Of importance, as several other S100 proteins, S100A4 is secreted by various cancer entities into the extracellular space and the blood 2, 6. This finding predestines S100A4 to be considered as a potential prognostic cancer biomarker 2. This has been also intensively discussed for melanoma, however, the functional importance of extracellular S100A4 has not been clearly established in this cancer 7. In breast cancer, to give one example of an epithelial cancer, extracellular S100A4 decreases cell adhesion and increases both cell migration and invasion 8.

Among natural targets of extracellular S100A4, RAGE and its downstream signalling pathways gained significant importance and, consequently, forms the link between those recent findings mentioned above 9. In colorectal cancer, S100A4 causes increased cell motility, which is mediated *via* the receptor for advanced glycation endproducts (RAGE) and downstream (mitogen‐activated protein kinase) (MAPK/ERK) signalling 10. Logically, knock down of S100A4 resulted in decreased metastasis formation in a xenografted mouse model of colorectal cancer 11. Very recently, the same group confirmed a similar role of S100A4 in thyroid cancer cells 12. Besides MAPK‐signalling pathways also NF‐κB‐dependent target genes represent potential candidates as mediators of S100A4‐stimulated tumour progression and metastasis in various epithelial and mesenchymal tumour cell lines 13.

Receptor for advanced glycation endproducts was detected clearly in human melanoma cells (G431 and A375 cells) but hardly in melanocytes 14. Recently, Wagner *et al*. confirmed that RAGE expression was elevated in melanoma tissue compared to benign nevi. They also suggested that RAGE signalling influences the process of melanoma development 15.

Meghnani *et al*. demonstrated that RAGE overexpression resulted in increased migration but decreased cell proliferation in WM115 primary human melanoma cells 16. In a preclinical experiment using mice xenografted with WM115 cells the same authors demonstrated that RAGE overexpression accompanied by increased levels of various RAGE ligands, among them S100A4, resulted in enhanced tumour growth *in vivo* 16. From their experiments, involving blockade of ligand‐RAGE interaction using anti‐RAGE antibodies, these authors suggested that RAGE and probably several S100 proteins are involved in melanoma growth.

However, data on the interaction of extracellular S100A4 with RAGE and its influence on the metastatic capacity of human melanoma cells are scarce. In own investigations recently we demonstrated interaction of recombinant human S100A4 with RAGE to be involved in NF‐κB activation and increased cytokine production in melanoma cells 4, 17. In consequence, we suggested that interaction of extracellular S100A4 with RAGE contributes to prometastatic activation of melanoma. Therefore, the present study aimed at a detailed discrimination of S100A4 secretion pathways in the human melanoma cell line A375 as model. Moreover, we examined the influence of overexpression and increased secretion of S100A4 as well as of S100A4‐RAGE interaction on *in vitro* cell motility, adhesion, migration and invasion.

## Materials and methods

### Cell culture

The human melanoma cell lines A375 and A2058 (purchased from ATCC, CRL‐1619, CRL‐1147), A375‐hRAGE 18 and MEL‐JUSO (purchased from DSMZ, ACC‐74) were cultured and cell extracts were prepared as published elsewhere 4.

### RNA preparation and PCR

Total RNA was isolated using miRNeasy Mini Kit (Qiagen, Hilden, Germany). RNA was treated with RNase‐free DNase (Fermentas, St. Leon‐Roth, Germany) to remove genomic DNA contamination. Reverse transcription and quantitative real‐time PCR were carried out in one step from 100 ng of RNA using QuantiTect SYBR Green RT‐PCR Kit (Qiagen). PCR conditions have been described previously 19. Following primers were used: human S100A4 forward (5′‐GGTGTCCACCTTCCACAAGT‐3′) and reverse (5′‐TGCAGGACAGGAAGACACAG‐3′), human β‐actin forward (5′‐GGACTTCGAGCAAGAGATGG‐3′) and reverse (5′‐AGCACTGTGTTGGCGTACAG‐3′). Human β‐actin was used as housekeeping gene to compare mRNA levels between different cell lines. Expression levels were calculated using 2^−ΔCt^, where ΔC_t_ was C_t_ value (threshold cycle) for S100A4 gene subtracted from C_t_ value of β‐actin in that sample.

### Construction of expression vectors and transfection

For generating stably transfected A375 cells, human cDNA of S100A4 was cloned into the mammalian expression vector pIRES2‐AcGFP1 (Clontech, Saint‐Germain‐en‐Laye, France). Briefly, the coding region of S100A4 was amplified by PCR using a 5′ oligonucleotide primer: 5′‐CCTTCTGCAGGCTGTCAT‐3′, containing PstI site (underlined) and a 3′ primer: 5′‐CATCAGAGGATCCTTCATTT‐3′, containing BamHI site (underlined). The amplified DNA was cut with restriction enzymes and ligated into the PstI and BamHI cloning sites of pIRES2‐AcGFP1. The pIRES2‐AcGFP1‐*hS100A4* plasmid construct was purified with a plasmid isolation kit (5 Prime, Hamburg, Germany), and transfected into A375 cells using Lipofectamine^™^ (Invitrogen, Darmstadt, Germany) according to manufacturer's instructions. Transfectants, termed as A375‐hS100A4, were selected in medium supplemented with 1.2 mg/ml G418 (Biochrom, Berlin, Germany). Transfected and wild‐type A375 cells used in this study were characterized by DNA profiling (Cell Line DNA Typing Report; DDC Medical, London, UK).

### Cellular growth *in vitro* and *in vivo*

To characterize growth behaviour of the novel established A375‐hS100A4 cell line *in vitro* and *in vivo* experiments were performed. Therefore, A375 and A375‐hS100A4 cells were seeded at a density of 1 × 10^5^ per well in a 6‐well plate and cultured for 5 days. Proliferative growth *in vitro* was estimated by counting the total number of living cells using a Casy Model TT cell counter (Roche, Mannheim, Germany). Moreover, both wild‐type A375 and transfected A375‐hS100A4 cells were used in a pilot experiment to establish melanoma xenograft models in NMRI (*nu/nu*) mice using a protocol published elsewhere with some modifications 20. In brief, single‐cell suspensions of both cell lines each with 5 × 10^6^ cells in 100 μl phosphate‐buffered saline were subcutaneously injected into the right upper flank of the mice. Lengths and widths of the tumours were measured twice a week using a sliding caliper. Tumour volumes were calculated by using the formula $\frac{\pi }{6}$ × length × width^2^. Mice were killed at day 23. Animal experiments were carried out according to the guidelines of the German Regulations for Animal Welfare. The protocol was approved by the local Ethical Committee for Animal Experiments (reference number 24‐9168.11‐4/2012‐1).

### SDS‐PAGE and Western blotting

S100A4 was detected *via* Western blotting as reported previously 4. Membranes were incubated with primary antibodies anti‐human S100A4 (DAKO, Hamburg, Germany) or anti‐RAGE (N‐16; Santa Cruz Biotechnology, Heidelberg, Germany) or anti‐β‐actin (Sigma‐Aldrich, Munich, Germany) and with corresponding secondary horseradish peroxidase‐conjugated antibodies (Sigma‐Aldrich). Optimal enhanced chemiluminescence (ECL) exposure times for cell lysates were adjusted for sensitive detection and optimal signal‐to‐noise ratio of both weak and strong signals depending on different protein expression levels. Densitometric analysis of Western blots was performed with TotalLab software (Total Lab Limited, New Castle upon Tyne, UK) as described elsewhere 21.

### Preparation of extracellular S100A4 from cell culture supernatants

Cells were seeded at 1 × 10^6^ per well in a 6‐well plate (Greiner Bio‐One, Frickenhausen, Germany) and incubated with 10% foetal calf serum (FCS)‐DMEM over night. After medium was removed, cells were washed with PBS and incubated with 0.1% FCS‐DMEM without any additives or with 0.2 μM bafilomycin A1, 3.6 μM brefeldin A (BFA), 10.4 μM cytochalasin B and 6.6 μM nocodazole (Sigma‐Aldrich) respectively. After incubation (4 or 8 hrs) cell culture supernatant (1000 μl) was obtained and centrifuged at 16,000 × *g* for 15 min. Afterwards, 950 μl of supernatant were discarded. The remaining volume was supplemented with sample buffer and denatured at 99°C for 10 min. These concentrated samples of cell culture supernatants were used for Western blot analysis to detect extracellular S100A4. Of importance, equal volumes of supernatants, which were standardized to the same number of cells per ‘inhibition’ sample, were loaded. Lactate dehydrogenase (LDH) activity was measured using the cytotoxicity detection kit (Roche) and applied according to manufacturer's instruction.

### ELISA

Concentration of extracellular S100A4 was determined by CircuLex^™^ S100A4 ELISA Kit (CycLex, MBL International, Woburn, MA, USA) and was performed according to manufacturer's instructions.

### S100A4 siRNA‐transfection

Small interfering RNAs (siRNAs) for silencing of the S100A4 gene (sc‐106781), siRNA‐transfection reagent, and medium were purchased from Santa Cruz Biotechnology and applied according to manufacturer's instructions. Two days after transfection, experiments were carried out after confirming successful transfection by Western blotting.

### Activation of NF‐κB p65 by extracellular S100A4

To examine the influence of extracellular S100A4 on NF‐κB p65 activation A375 and A375‐hS100A4 cells were cultured with complete media. Then, culture medium of A375‐hS100A4 cells was removed, centrifuged at 500 × *g* for 5 min. and added to A375 cells for 24 hrs. To prepare nuclear extracts cells were lysed in 150 μl hypotonic lysis buffer (100 mM HEPES (4‐(2‐hydroxyethyl)‐1‐piperazineethanesulfonic acid) pH 7.9, 15 mM MgCl_2_, 100 mM KCl, 1 mM DTT, 1 mM phenylmethyl sulfonyl fluoride and 10 μg/ml leupeptin) and incubated for 15 min. at 4°C. After addition of 7.5 μl 10% Nonidet P40 and centrifugation at 15,000 × *g* for 5 min. at 4°C, pellets were resuspended in 50 μl extraction buffer (20 mM HEPES pH 7.9, 1.5 mM MgCl_2_, 0.42 M NaCl, 0.2 mM ethylenediaminetetraacetic acid, 25% glycerin, 1 mM DTT, 1 mM phenylmethyl sulfonyl fluoride and 10 μg/ml leupeptin), incubated with shaking for 30 min. at 4°C and centrifuged at 15,000 × *g* for 15 min. at 4°C. NF‐κB activation was measured from 10 μl of the supernatant containing nuclear proteins using an ELISA kit (NF‐κB p65 Transcription Factor Kit, Thermo Fisher Scientific, Bonn, Germany) according to manufacturer's protocol.

### Adhesion assay

The adhesive properties of A375 and A375‐hS100A4 cells were analysed by adhesion experiments on fibronectin as described previously 22.

### Scratch assay

Cells were seeded in 6‐well plates to reach 100% confluence within 24 hrs (1 × 10^6^ cells/well). A vertical scratch was generated in the cell monolayer using a sterile micropipette tip 22. Cells were washed with PBS and 0.1% FCS‐DMEM was added to impair cell proliferation. Cells were cultivated under normal conditions and images of the wound closure were captured after 24 hrs 22. For further investigations scratch assays were carried out in the presence of 3.6 μM BFA, recombinant S100A4 4 or soluble RAGE (sRAGE) 18 in fivefold (in A375 cells; 150 ng/ml S100A4 or sRAGE) or 100‐fold molar excess (in A375‐hRAGE cells; 3000 ng/ml S100A4) than extracellular S100A4 determined by ELISA, and blocking Anti‐RAGE antibody (10 μg/ml; R&D, Wiesbaden, Germany).

### Migration and invasion assay

For migration and invasion experiments, the upper side of a Boyden chamber PET membrane with 8‐μm pores (Thincert^™^ 24 well cell culture insert, Greiner Bio‐One, Frickenhausen, Germany) either was left uncoated (migration) or was coated (invasion) with Matrigel (2.5 mg/ml, BD Matrigel^™^ Basement Membrane Matrix Growth Factor Reduced, Phenol Red Free, BD Biosciences, Heidelberg, Germany) for 30 min. at 37°C. Prior to experiment, A375, A375‐hS100A4 and A375‐hRAGE cells were stained with calcein AM (BD Biosciences, Bedford, MA, USA) or CellTracker^™^ Green (Invitrogen). Assays were performed without addition of FCS to impair cell proliferation. Further assay procedures were described previously 22. Furthermore, cells were incubated with either recombinant S100A4 or sRAGE (see Scratch assay).

### Statistical analysis

Descriptive data were expressed as mean ± S.E.M. Nonparametric statistical analyses (Wilcoxon–Mann–Whitney test) were calculated by using the SPSS 20 (IBM, Ehningen, Germany) software package. For all analyses a value of *P* < 0.05 was considered as statistically significant.

## Results

### Expression and synthesis of S100A4 and RAGE

**Figure 1:**
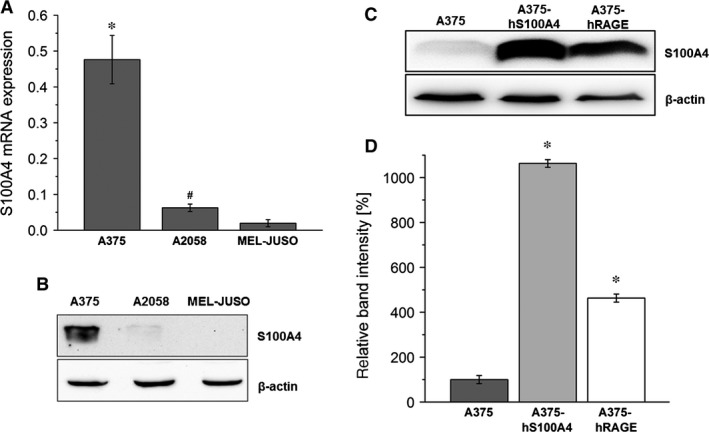
Expression and synthesis of S100A4. (**A**) Relative mRNA expression of S100A4 in A375, A2058 and MEL‐JUSO cell lysates was analysed by quantitative real‐time RT‐PCR. Displayed are the 2^−ΔCt^ values, representing the S100A4 gene expression normalized to the β‐actin endogenous reference gene (mean ± S.E.M., *n* ≥ 6, *P < 0.05, *versus* A2058 and MEL‐JUSO, ^#^ *P* < 0.05, *versus* MEL‐JUSO). Representative Western blots show S100A4 protein synthesis in wild type A375, A2058 and MEL‐JUSO cell lysates (**B**) and in wild type A375, A375‐hS100A4, and A375‐hRAGE cell lysates (**C**). Exposition time of S100A4 blot was 1 min. (**C**) up to 10 min. (**B**). β‐actin was used as loading control. (**D**) Densitometric analysis of S100A4 expression in wild type and transgenic A375 cells related to β‐actin expression (mean ± S.E.M., *n* = 5, *P < 0.05, *versus* A375).

**Figure 2:**
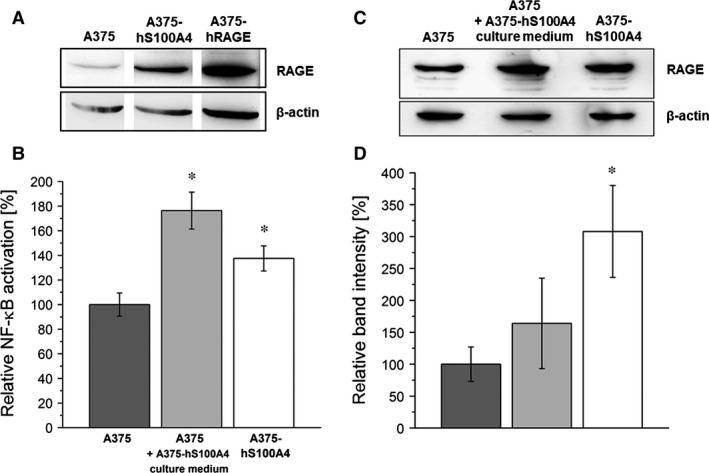
Synthesis of RAGE and S100A4‐mediated NF‐κB p65 activation in melanoma cells. (**A**) Representative Western blot shows RAGE protein synthesis in A375, A375‐hS100A4, and A375‐hRAGE cell lysates. β‐actin was used as loading control. (**B**) NF‐κB p65 activation in nuclear extracts of wild type A375, wild type A375 cells re‐treated with culture medium of A375‐hS100A4 cells, and A375‐hS100A4 cells (mean ± S.E.M., *n* = 4). (**C**) Representative Western blot shows S100A4‐mediated up‐regulation of RAGE protein synthesis in wild type A375 cells re‐treated with culture medium of A375‐hS100A4 cells and A375‐hS100A4 cells compared to wild type A375 cells. β‐actin was used as loading control. (**D**) Densitometric analysis of RAGE expression in wild type A375, wild type A375 cells re‐treated with culture medium of A375‐hS100A4 cells, and A375‐hS100A4 cells related to β‐actin expression (mean ± S.E.M., *n* = 3, *P < 0.05, *versus* A375).

Expression of S100A4 and RAGE was investigated in three different human melanoma cell lines (A375, A2058, and MEL‐JUSO) well‐established in many laboratories. In all cell lines S100A4 mRNA was detectable showing different expression levels (Fig. 1A). A375 cells showed a significantly higher mRNA expression of S100A4 compared to A2058 and MEL‐JUSO cells. Synthesis of S100A4 was determined using Western blotting (Fig. 1B). In cell extracts of A2058 and MEL‐JUSO S100A4 protein was hardly or not verifiable. Substantial S100A4 synthesis was only detected in A375 cells. Therefore, this cell line was selected for stable overexpression of S100A4 to study the impact of S100A4 on prometastatic processes in more detail. A375 cells stably transfected with pIRES2‐AcGFP1‐hS100A4, termed A375‐hS100A4, displayed a significantly increased S100A4 synthesis compared to wild‐type A375 cells (Fig. 1C). Densitometric analysis revealed a 10‐fold higher S100A4 expression in A375‐hS100A4 cells compared to wild‐type A375 cells (Fig. 1D). To further elucidate an involvement of RAGE in S100A4 signalling, we investigated S100A4 synthesis in RAGE overexpressing A375 cells, termed A375‐hRAGE. As expected, transgenic A375‐hRAGE cells showed highest RAGE synthesis of the cell lines investigated (Fig. 2A). Interestingly, these cells also synthesized fivefold higher levels of S100A4 compared to wild‐type A375 cells (Fig. 1D). Conversely, A375‐hS100A4 cells also showed a higher expression of RAGE compared to wild‐type cells (Fig. 2A). Thus, S100A4 clearly seemed to act as an autocrine regulator in these cells. This finding was functionally confirmed by evaluation of NF‐κB p65 activation (Fig. 2B). Therefore, A375 cells were re‐treated with cultivated medium of A375‐hS100A4 cells. After 24 hrs the re‐treated A375 and the A375‐hS100A4 cells showed a significantly increased NF‐κB p65 activation compared to untreated A375 cells. RAGE synthesis was re‐evaluated in this experimental setting, revealing higher RAGE levels in re‐treated A375 and A375‐hS100A4 cells compared to A375 wild‐type cells (Fig. 2C and D).

### Detection of extracellular S100A4

**Figure 3:**
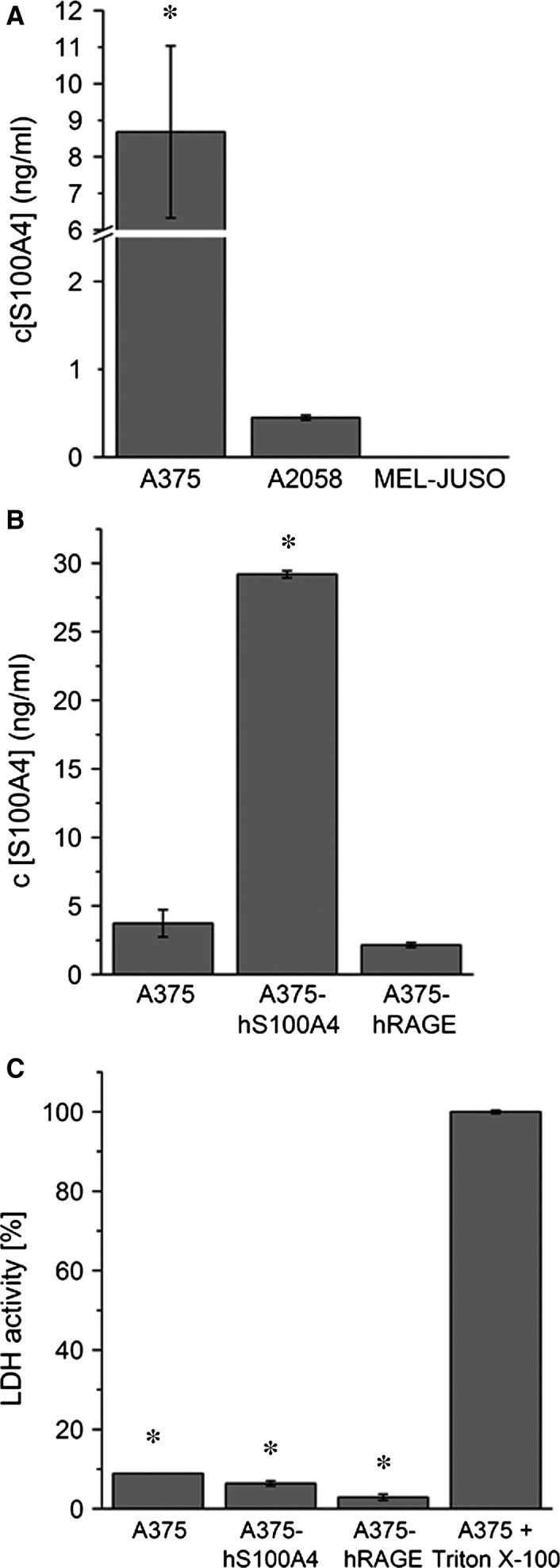
Active secretion of S100A4. (**A**) Extracellular S100A4 was detected after 4 hrs of incubation in cell culture supernatants of A375, A2058, and MEL‐JUSO cells *via* ELISA (mean ± S.E.M., *n* = 3, *P < 0.05, *versus* A2058 cells). (**B**) Detection of extracellular S100A4 in cell culture supernatants of A375, A375‐hS100A4, and A375‐hRAGE cells showing highest amounts of S100A4 in supernatant of A375‐hS100A4 cells after 8 hrs of incubation (mean ± S.E.M., *n* = 3, *P < 0.05, *versus* A375 cells). (**C**) Detection of LDH activity in cell culture supernatants of A375, A375‐hS100A4, and A375‐hRAGE cells. Incubation with medium was set as 0% LDH activity and incubation with Triton X‐100 was set as 100% (mean ± S.E.M., *n* = 3, *P < 0.01, *versus* Triton X‐100).

**Figure 4:**
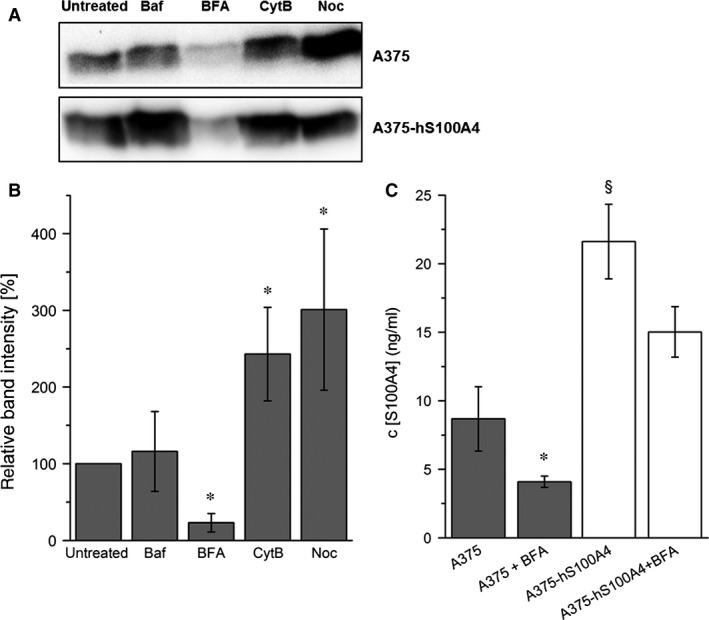
Identification of secretion pathway of S100A4. (**A**) Representative Western blots show detection of extracellular S100A4 in concentrated samples of cell culture supernatants of A375 and A375‐hS100A4 cells analysed untreated or after incubation with bafilomycin A1 (Baf), brefeldin A (BFA), cytochalasin B (CytB), or nocodazole (Noc) after 4 hrs of incubation. (**B**) Densitometric analysis of Western blots showing detection of extracellular S100A4 in A375 cells after 4 hrs incubation with different inhibitors related to β‐actin expression (mean ± S.E.M., *n* = 5, *P < 0.05, *versus* untreated). (**C**) Untreated and BFA‐treated A375 and A375‐hS100A4 cells were incubated for 4 hrs and concentration of extracellular S100A4 in cell culture supernatants were measured *via* ELISA (mean ± S.E.M., *n* = 3, *P < 0.05, *versus* untreated; ^§^ *P* < 0.05, *versus* A375 cells).

S100A4 secretion in wild‐type melanoma cells (Fig. 3A) and transgenic A375 cells (Fig. 3B) was demonstrated showing highest amounts of S100A4 concentration in A375‐hS100A4 cells. In wild‐type melanoma cells, S100A4 secretion correlates well with S100A4 expression/synthesis. Active secretion was confirmed by measurement of LDH. Presence of disrupted cells in the samples was excluded by measuring significantly lower LDH activity in all cell culture supernatants of untreated cells (4–9%, *P* < 0.01) compared to maximum releasable LDH activity in those cells treated with Triton X‐100 (100%; Fig. 3C). As expected, A375‐hS100A4 cells secreted significantly higher amounts of S100A4 compared to wild‐type A375 cells after 8 hrs (29 ng/ml *versus* 4 ng/ml; Fig. 3B) and also after 4 hrs (22 ng/ml *versus* 8 ng/ml; Fig. 4C). Interestingly, S100A4 secretion measured in A375‐hRAGE cell supernatants showed lower amounts of free S100A4 (2 ng/ml) compared to those measured in wild‐type A375 cell supernatants despite higher S100A4 amounts in A375‐hRAGE cell lysates (*versus* A375 cells). The latter indicates that extracellular S100A4 in part could have been RAGE‐bound, which also is indicative to the above mentioned autocrine regulation of these cells.

### Secretion pathway of S100A4

To characterize the pathway of active S100A4 secretion in A375 cells in more detail cells were incubated for 4 hrs with various well‐established inhibitors. Bafilomycin A1, a blocker of vacuolar H^+^‐ATPase, was used to identify the type of vesicles carrying the proteins 23. Brefeldin A inhibits the conventional endoplasmatic reticulum (ER)‐Golgi secretion pathway 24, 25. For inhibition of unconventional pathways cytochalasin B was used to block actin filament‐dependent pathways 26, whereas treatment with nocodazole was used to depolymerize microtubules and restrain tubulin‐dependent routes 27. Secretion of S100A4 in A375 and A375‐hS100A4 cells was inhibited after addition of BFA essentially indicating the classical ER–Golgi pathway involved in S100A4 secretion (Fig. 4A). A significant decrease in S100A4 concentration after 4 hrs of incubation with BFA was also confirmed by densitometric analysis and ELISA in A375 cells (Fig. 4B and C). In A375‐hS100A4 cells concentration of S100A4 was only slightly diminished after incubation with BFA as a result of S100A4 overexpression in these cells. Interestingly, after treatment with cytochalasin B and nocodazole higher amounts of S100A4 were found in cell culture supernatants of A375 cells (Fig. 4B). Possibly, this was because of the depolymerizing effects on microtubules and actin filaments that might not only destroy the filaments for protein transport but also those for the cell structure resulting in an altered cell shape and a disruption of the cells leading to a passive protein release.

### Influence of S100A4 overexpression on growth and prometastatic activation

**Figure 5:**
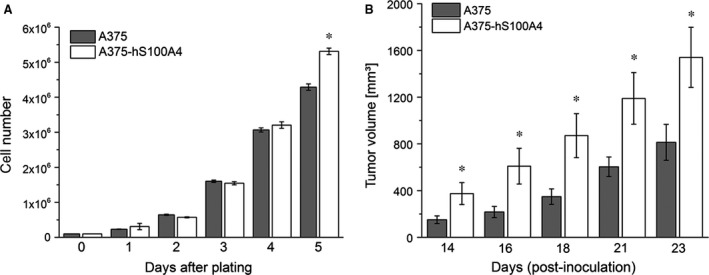
Influence of S100A4 overexpression on cellular growth *in vitro and in vivo*. (**A**) Cell growth was evaluated for wild type A375 and A375‐hS100A4 cells (mean ± S.E.M., *n* ≥ 9, *P < 0.05, *versus* wild type A375). (**B**) To assess tumour growth A375 and A375‐hS100A4 cells were injected subcutaneously into the right upper flank of female NMRI (*nu/nu*) mice. The average volume of the tumours was measured for a period of three weeks (mean ± S.E.M., *n* ≥ 9, *P < 0.05, *versus* wild type A375).

**Figure 6:**
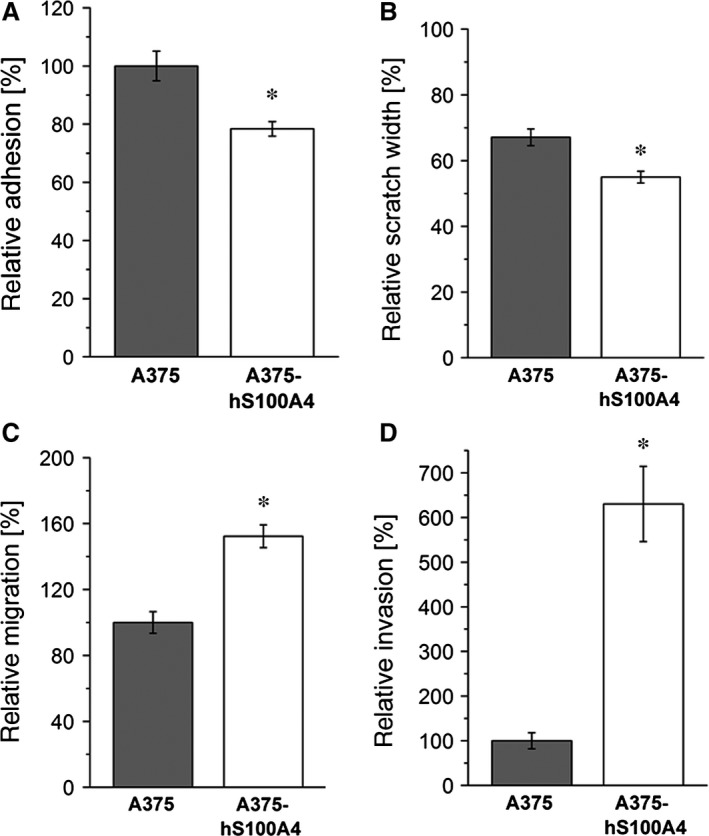
Effect of S100A4 overexpression on cell adhesion, motility, migration, and invasion in A375 cells. Relative adhesion (**A**), relative scratch widths (**B**), relative migration (**C**), or relative invasive capability (**D**) of A375 and A375‐hS100A4 cells was measured after 24 hrs. Adhesion, migration, and invasion rate of untreated control cells were set as 100%. Initial scratch width was set as 100% (mean ± S.E.M., *n* = 3, *P < 0.05, versus A375 cells).

First, we investigated the influence of S100A4 overexpression on cellular growth *in vitro* and *in vivo*. Here, higher concentrations of S100A4 increased cell growth as well as tumour growth after 5 or 14 days respectively (Fig. 5). Next, we analysed the effect of S100A4 overexpression on adhesion, motility, migration and invasion, cellular properties strongly suggested to be involved in metastasis. Figure 6A displays adhesion to fibronectin showing a significantly reduced adhesion of A375‐hS100A4 cells by approximately 20% compared to A375 cells. As shown in Figure 6B, motility of A375‐hS100A4 cells in scratch assays was significantly increased in comparison with A375 cells (45% *versus* 33%). Boyden chamber assays revealed a significant increment in both the migratory (Fig. 6C) and invasive (Fig. 6D) behaviour of A375‐hS100A4 cells. Here, cells displayed a 1.5‐fold increased migratory activity and even a sixfold increased invasive capability compared to A375 cells.

### Influence of S100A4‐RAGE interaction on motility, migration and invasion

**Figure 7:**
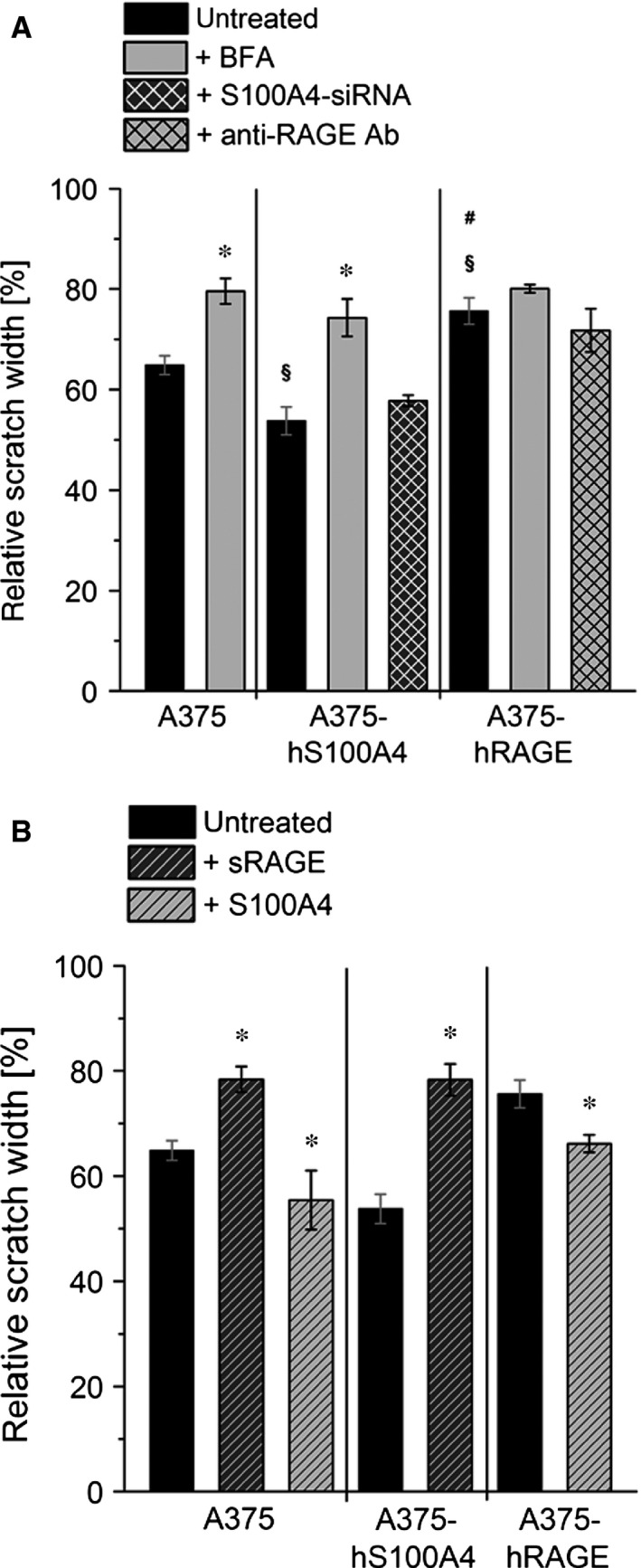
Regulation of cell motility by S100A4‐RAGE interaction. Relative scratch widths of wild type A375, A375‐hS100A4, and A375‐hRAGE cells either (**A**) untreated, treated with BFA, S100A4‐siRNA, and blocking Anti‐RAGE antibody, or (**B**) treated with fivefold molar excess (150 ng/ml) of sRAGE, fivefold (150 ng/ml; in A375 cells) or 100‐fold molar excess (3000 ng/ml) of S100A4 (in A375‐hRAGE cells) are shown. Initial scratch width was set as 100% (mean ± S.E.M., *n* ≥ 3, *P < 0.05, *versus* untreated; ^§^ *P* < 0.05, *versus* untreated A375 cells; ^#^ *P* < 0.05 *versus* untreated A375‐hS100A4 cells).

We further examined whether blocking of S100A4 secretion through BFA, gene silencing by siRNA, or, on the other hand, addition of either recombinant S100A4 or recombinant sRAGE will influence prometastatic effects of S100A4. Therefore, we performed scratch assays with untreated A375 cells in comparison with both BFA‐treated and siRNA‐transfected A375‐hS100A4 cells (Fig. 7A). After incubation with BFA, motility significantly decreased in wild‐type A375 cells (by 26%) and transgenic A375‐hS100A4 cells (by 21%) compared to untreated controls (Fig. 7A). In BFA‐treated A375‐hRAGE cells motility tendentially was also lowered (by 20%) compared to untreated controls. Since blocking with BFA is not selective for S100A4 secretion, we transfected A375‐hS100A4 cells with siRNA directed against S100A4 mRNA, termed as A375‐siS100A4. As expected both S100A4 synthesis and S100A4 secretion were down‐regulated (Fig. S1). A375‐siS100A4 cells showed a lower motility compared to A375‐hS100A4 cells (43% *versus* 47%) but this effect did not reach statistical significance. As an explanation, in siRNA experiments both intracellular and extracellular S100A4 is affected. Therefore, unknown compensatory mechanisms resulting from lower intracellular S100A4 levels are assumed to affect cell processes. As S100A4 was reported to influence, for example, cytoskeletal regulation and reorganization of tubuli 28, 29, it can be deduced that a loss of S100A4 has to be compensated to maintain cellular properties. Blockade of ligand binding to RAGE by using an anti‐RAGE antibody only slightly diminished cell motility. To demonstrate an interaction between S100A4 and RAGE, we incubated cells either with recombinant S100A4 or with sRAGE. Here, in contrast to the effects of siRNA‐transfection, only extracellular concentration of S100A4 is affected. Addition of fivefold molar excess of S100A4 to wild‐type A375 cells resulted in a significantly increased motility (Fig. 7B). In A375‐hRAGE cells a 100‐fold molar excess of S100A4 (compared to initial S100A4 concentration measured in cell culture supernatants of A375‐hS100A4) also significantly increased motility. Addition of fivefold molar excess of sRAGE significantly diminished motility of A375 and A375‐hS100A4 cells (Fig. 7B). In this regard, former experiments revealed that molar excess of sRAGE effectively inhibits the interaction of membrane RAGE with S100 ligands 18.

**Figure 8:**
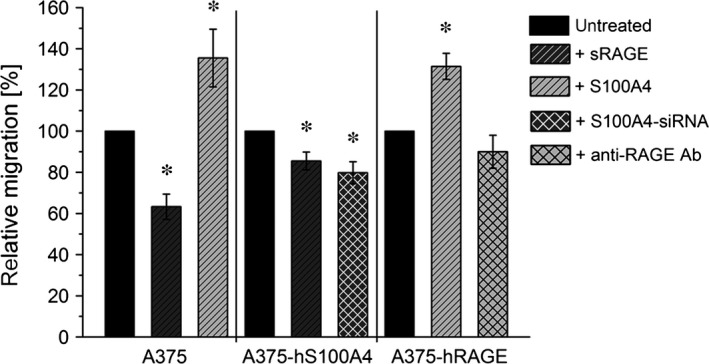
Regulation of cell migration by S100A4‐RAGE interaction. Relative migration of wild type A375, A375‐hS100A4, and A375‐hRAGE cells (untreated, fivefold molar excess (150 ng/ml) of sRAGE, fivefold (150 ng/ml; in A375 cells) or 100‐fold molar excess (3000 ng/ml) of S100A4 (in A375‐hRAGE cells), S100A4‐siRNA, and blocking Anti‐RAGE antibody) are shown. Migration rate of untreated control cells were set as 100% (mean ± S.E.M., *n* ≥ 3, *P < 0.05, *versus* untreated).

**Figure 9:**
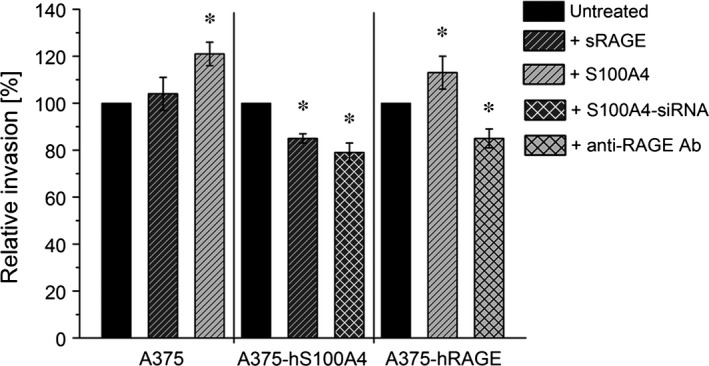
Regulation of cell invasion by S100A4‐RAGE interaction. Relative invasion of wild type A375, A375‐hS100A4, and A375‐hRAGE cells (untreated, fivefold molar excess (150 ng/ml) of sRAGE, fivefold (150 ng/ml; in A375 cells) or 100‐fold molar excess (3000 ng/ml) of S100A4 (in A375‐hRAGE cells), S100A4‐siRNA, and blocking Anti‐RAGE antibody) are shown. Invasion rate of untreated control cells were set as 100% (mean ± S.E.M., *n* ≥ 3, *P < 0.05, *versus* untreated).

Furthermore, we investigated the migratory behaviour of A375, A375‐hS100A4 and A375‐hRAGE cells. A375 cells incubated with sRAGE migrated significantly slower (only 63%) compared to untreated cells (100%), whereas addition of S100A4 significantly increased migration by 36% (Fig. 8). In A375‐hS100A4 cells, incubation with sRAGE significantly decreased migration by 24%. Of importance, transfection with siRNA significantly decreased migration by 20%. Additionally, in A375‐hRAGE cells, incubation with S100A4 resulted in a significantly increased migration by 23%, whereas blockade of ligand binding to RAGE by using an anti‐RAGE antibody 30 decreased migration by 10% but showed no statistical significance. On the other hand, investigations concerning invasive capability confirmed the stimulating effect of S100A4 (Fig. 9). Addition of S100A4 in A375 cells showed a significantly increased invasion by 21%, whereby after addition of sRAGE in A375‐hS100A4 cells invasion was significantly diminished by 15%. Furthermore, transfection with siRNA significantly decreased invasion by 21%. In A375‐hRAGE, addition of S100A4 significantly increased invasion by 13% and usage of blocking antibody significantly decreased invasion by 15%.

## Discussion

S100A4 is reported to play a key role in development of advanced cancer stages including investigations in tumour xenograft and genetically engineered mouse models 3, 31, 32. However, the activity of extracellular S100A4 within the metastatic cascade, and the participation of S100A4 in malignant melanoma are still not fully understood.

In this study an association of elevated extracellular S100A4 and a prometastatic phenotype in melanoma cells could be demonstrated. For this purpose, the highly metastatic A375 melanoma cell line was chosen as a model because of its comparably high baseline expression and synthesis of S100A4. We demonstrated that in A375 cells S100A4 is secreted *via* the classical ER–Golgi pathway. The observation of a classical, BFA‐sensitive, ER–Golgi pathway‐dependent secretion of S100A4 in melanoma cells, first of all, contributes to further understanding of the enormous cell‐ and protein‐specific metabolic heterogeneity of the S100 family. This heterogeneity is impressively reflected by the fact that even melanoma cell lines among themselves seem to exhibit different secretion pathways for the same protein. In line with this, our observation is more a basic finding on one commonly used specific melanoma cell line than a generalizable finding with a prompt specific clinical relevance in melanoma.

Of importance, overexpression of S100A4 was accompanied by RAGE up‐regulation and, vice versa, overexpression of RAGE was accompanied by an S100A4 up‐regulation indicating S100A4 to act as an autocrine regulator. Meghnani and colleagues also reported an up‐regulation of the RAGE ligands S100B, S100A2, S100A4, S100A6 and S100A10 in RAGE overexpressing tumours 16. Furthermore, we demonstrated that S100A4 overexpression led to an increased cell growth *in vitro* as well as to faster tumour growth *in vivo* which is also consistent with data reported by Meghnani *et al*. 16.

Interaction of different S100 proteins with RAGE is considered to be cell‐ and tissue‐specific depending on the S100 protein that is preferentially synthesized by a tumour entity 7, 9. In this regard, recent papers demonstrated that S100A7 promotes migration and invasion *via* RAGE in osteosarcoma cells as well as in a squamous carcinoma cell line 33, 34. Although other studies reported a RAGE‐independent signalling of S100 proteins 35, this study gives further evidence for a RAGE‐dependent mechanism in melanoma cells. Contributing to this we previously supposed that activation of melanoma cells by S100 proteins takes place by RAGE downstream signalling activation of NF‐κB as we demonstrated an increased NF‐κB activation caused by the treatment with recombinant S100A4 17. Very recently, we demonstrated an increased invasiveness and NF‐κB activation in B16‐F10 mouse melanoma cells by macrophage‐derived S100A4 36. Chaabane and colleagues reported an NF‐κB activation in a RAGE‐dependent manner caused by S100A4‐rich conditioned medium in smooth muscle cells 37.

To support our *in vitro* observations that especially extracellular S100A4 takes influence within the metastatic cascade, we showed that enhanced motility properties could be diminished by inhibition of the secretory pathway. Moreover, in A375‐hS100A4 cells specific silencing of S100A4 by RNA interference resulted in a decreased effect on prometastatic processes. However, the effects observed in siRNA experiments were smaller compared to those originated by treatment with BFA. BFA only blocks the secretion of S100A4, while the intracellular S100A4 content remains nearly unaffected. In siRNA experiments both intracellular and extracellular S100A4 are affected. Therefore, compensatory or modulated mechanisms resulting from lower intracellular S100A4 levels are assumed to affect cellular processes, possibly explaining the differences observed. Furthermore, using a blocking antibody against RAGE led to minor effects on cell motility, migration and invasion. However, usage of a blocking antibody against such a multiligand receptor also affects other signalling pathways leading to alternative signalling routes. Recently, Hansen and colleagues showed, that S100A4 might also mediate its function through binding to TLR4 38. Therefore, blocking of RAGE might cause alternative binding sites of S100A4 in these cells. This also has to be considered when interpreting the results of blocking experiments reported by Meghnani *et al*. 16 and others as discussed above.

In summary, high concentrations of extracellular S100A4 stimulated prometastatic effects, whereas reduction of extracellular S100A4 concentration by sRAGE reduced prometastatic processes. Extracellular sRAGE binds to S100A4 and inhibits ligand binding to membrane RAGE. Consequently, downstream signalling cascades are not activated and motility, migration and invasion rates are diminished.

In conclusion, active secretion of S100A4 and its subsequent interaction with coexpressed RAGE in an autocrine manner is suggested to be an important prometastatic attribute in those melanoma cases characterized by a high secretion rate of S100A4. This possibly provides additional molecular targets for novel therapeutic approaches aiming at blockade of ligand binding to RAGE or RAGE signalling to inhibit melanoma metastasis.

## Conflicts of interest

The authors state no conflict of interest.

## Supporting information


**Figure S1** Synthesis and secretion of S100A4 after transfection with siRNA.
