# Supplementary material for: Interaction of extracellular S100A4 with RAGE prompts prometastatic activation of A375 melanoma cells
Source: J Cell Mol Med. 2016 Mar 1;20(5):825–35. doi: 10.1111/jcmm.12808 (PMC4831350; doi:10.1111/jcmm.12808)
Supplement: Supplementary file 1 — Figure S1 Synthesis and secretion of S100A4 after transfection with siRNA. [file JCMM-20-825-s001.docx]

Supporting information


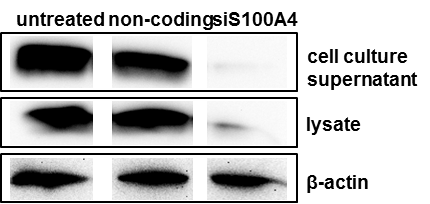


**Figure S1: Synthesis and secretion of S100A4 after transfection with siRNA**

Representative Western blots show detection of S100A4 in concentrated samples of cell culture supernatants and cell lysates of A375-hS100A4 cells before (untreated), after treatment with non-coding siRNA (non-coding), and after S100A4 gene silencing through RNA interference (siS100A4). β-actin was used as loading control.
